# Identification of two novel papillomaviruses in belugas

**DOI:** 10.3389/fmicb.2023.1165839

**Published:** 2023-07-26

**Authors:** Youyou Li, Meifang Xiao, Yun Zhang, Zihan Li, Shijie Bai, Haoxiang Su, Ruoyan Peng, Gaoyu Wang, Xiaoyuan Hu, Xinran Song, Xin Li, Chuanning Tang, Gang Lu, Feifei Yin, Peijun Zhang, Jiang Du

**Affiliations:** ^1^Hainan Medical University-The University of Hong Kong Joint Laboratory of Tropical Infectious Diseases, Key Laboratory of Tropical Translational Medicine of Ministry of Education, Hainan Medical University, Haikou, China; ^2^Department of Clinical Laboratory, Center for Laboratory Medicine, Hainan Women and Children’s Medical Center, Haikou, China; ^3^Marine Mammal and Marine Bioacoustics Laboratory, Laboratory of Marine Viruses and Molecular Biology, Institute of Deep-sea Science and Engineering, Chinese Academy of Sciences, Sanya, China; ^4^National Health Commission, Key Laboratory of Systems Biology of Pathogens, Institute of Pathogen Biology, Chinese Academy of Medical Sciences and Peking Union Medical College, Beijing, China; ^5^Dalian Sun Asia Tourism Holding Co., Ltd., Dalian, China; ^6^Qingdao Polar Haichang Ocean Park, Qingdao, China

**Keywords:** diversity, novel PVs, evolution, beluga whale, next-generation sequencing

## Abstract

**Introduction:**

Papillomaviruses (PVs) can cause hyperplasia in the skin and mucous membranes of humans, mammals, and non-mammalian animals, and are a significant risk factor for cervical and genital cancers.

**Methods:**

Using next-generation sequencing (NGS), we identified two novel strains of papillomavirus, PV-HMU-1 and PV-HMU-2, in swabs taken from belugas (*Delphinapterus leucas*) at Polar Ocean Parks in Qingdao and Dalian.

**Results:**

We amplified the complete genomes of both strains and screened ten belugas and one false killer whale (*Pseudorca crassidens*) for the late gene (L1) to determine the infection rate. In Qingdao, 50% of the two sampled belugas were infected with PV-HMU-1, while the false killer whale was negative. In Dalian, 71% of the eight sampled belugas were infected with PV-HMU-2. In their L1 genes, PV-HMU-1 and PV-HMU-2 showed 64.99 and 68.12% amino acid identity, respectively, with other members of *Papillomaviridae*. Phylogenetic analysis of combinatorial amino acid sequences revealed that PV-HMU-1 and PV-HMU-2 clustered with other known dolphin PVs but formed distinct branches. PVs carried by belugas were proposed as novel species under *Firstpapillomavirinae*.

**Conclusion:**

The discovery of these two novel PVs enhances our understanding of the genetic diversity of papillomaviruses and their impact on the beluga population.

## 1. Introduction

Papillomavirus (PV) can cause persistent infections in various parts of the skin and mucous membranes in humans and mammals, resulting in epidermal proliferative lesions (Cheng et al., 2023; Yang et al., 2023). Human genital PV (HPV) types are primarily responsible for cervical, vulvar, penile, and other anogenital cancers (Pino et al., 2023; Seyoum et al., 2023). Although papillomaviruses have been detected in all vertebrate populations except amphibians, most of them have been identified in humans and other mammals (Van Doorslaer et al., 2017). However, novel papillomaviruses have been recently detected in the cartilage of *Fulmarus glacialis* (Gaynor et al., 2015) and the liver of *Hemidactylus frenatus* (Agius et al., 2019) using next-generation sequencing (NGS). The increase in the number of viruses is associated with viruses that are typically found in terrestrial mammals, being found in marine mammals as well. For instance, Mäkelä et al. (2015) found respiratory diseases in marine mammals caused by influenza A and B virus infections (Mäkelä et al., 2015). Fouchier et al. (2004) found that H7N7 infected seals, which then infected human beings causing conjunctivitis (Fouchier et al., 2004). In addition, the US Centers for Disease Control and Prevention revealed that from 2007 to 2009, various belugas in North America were infected with the seal pox virus, which caused various diseases (Roess et al., 2011) and the individuals who were in close contact with belugas also tested positive for the virus. A study at the University of Florida found that the San Miguel sea lion virus (SMSV) was indistinguishable from the vesicular exanthema of swine virus (VESV), which caused vesicular inflammation in California pigs (Waltzek et al., 2012). These studies suggest the possibility of zoonotic viruses being carried by marine mammals and the risk of cross-species transmission. Cui et al. (2019) and Zhou et al. (2020) found that severe acute respiratory syndrome coronavirus (SARS-CoV) and Middle East respiratory syndrome coronavirus (MERS-CoV), two highly infectious and pathogenic viruses, might have originated from bats. The genetic recombination ability of coronaviruses makes them more adaptable to their hosts and increases their survival (Su et al., 2016). Therefore, the growing global virus diversity and potential spillovers require attention (Cui et al., 2019).

Papillomaviruses (PVs) are a diverse group of small, non-enveloped viruses belonging to the family *Papillomaviridae*, with double-stranded DNA genomes ranging in length from 5 to 8 kb (Zhang et al., 2022). The structurally conserved circular genome of PVs is organized into several partially overlapping open reading frames (ORFs) (Arman and Munger, 2022). These include the major genes present in all PVs: the early genes (E1 and E2), which are responsible for replication (Kraberger et al., 2022; Prabhakar et al., 2023), regulation of the life cycle, viral gene expression. The late genes (L1 and L2) are controlling the formation of viral capsids. Many PVs also carry other early genes, viral oncogenes E6 and E7 that are responsible for their ability to interfere with tumor suppressor genes such as p53 and the retinoblastoma protein (pRB) family (Nadile et al., 2022; Sofiani et al., 2023; Zhi et al., 2023). The upstream regulatory region or long control region (URR or LCR) located between the L1 and E6 ORFs is responsible for the origin of virus replication, as well as virus and cell transcription factor binding sites (TFBS) (Warburton et al., 2018; Coursey et al., 2021).

While the understanding of non-human PV types has increased in recent years, knowledge about the diversity of animal PVs remains limited (Bernard et al., 2010). The discovery of bovine and human papillomavirus sequences in cats suggests the possibility of cross-species transmission (Egberink et al., 2013). Recently, research on invertebrates and vertebrates in marine ecosystems has focused on the origin, evolution, and emergence of terrestrial viruses. Papillomaviruses were first reported in porpoises (*Phocoena phocoena*) (Gottschling et al., 2011) and subsequently found in many other small Odontoceti, including common dolphins (*Delphinus delphis*) (Robles-Sikisaka et al., 2012), common bottlenose dolphins (*Tursiops truncatus*) (Bossart et al., 2019), and burmester’s porpoises (*Phocoena spinipinnis*) (Van Bressem et al., 2007; Brimer et al., 2017). In order to expand our understanding of the diversity of marine animal PVs, we conducted a marine mammal virus survey program and discovered two novel papillomaviruses (PV-HMU-1 and PV-HMU-2) from nose, throat, and anal swab samples of belugas (*Delphinapterus leucas*). We obtained and characterized the complete genome sequences of PV-HMU-1 and PV-HMU-2. The alignment results of the novel PVs revealed low amino acid identities with other known papillomaviruses and clustered with dolphin papillomaviruses in the evolutionary tree. The findings of this study deepen our understanding of the host and genetic diversity of papillomaviruses and provide essential data for the possible zoonotic transmission caused by animal-origin PVs.

## 2. Materials and methods

### 2.1. Swab sample collection

We collected a total of 21 nose, throat, and anal swabs from ten belugas and one false killer whale (*Pseudorca crassidens*), housed in aquaria at Qingdao Polar Haichang Ocean Park and Dalian Sun Asia Tourism Holding Co., China in April 2018 (Supplementary Table 1). The nine throat, nose, and anal swabs from mammals in Qingdao constituted pool 49 (Table 1), while the 12 throat and anal swabs from Dalian constituted pool 41 (Table 2). The sampling procedures were approved by the Ethics Committee of the Hainan Medical University (Approval number: HMUEC20180059). The collected samples were quickly immersed in the maintenance medium in virus-sampling tubes (Yocon Biology, Beijing, China), to ensure sample quality, and transported to the laboratory within 24 h using an ice-cold dry chain (Wu et al., 2018a). The samples were then stored at −80°C (Wu et al., 2018a).

**TABLE 1:** Detection rate of PV-HMU-1 among belugas from Qingdao Ocean Park.

Numbers	Animal species	Throat swab	Nose swab	Anal swabs	Sampling date	Sampling place
1	*Delphinapterus leucas*	+	−	−	19 April, 2018	Qingdao
2	*D. leucas*	−	−	−	19 April, 2018	Qingdao
3	*Pseudorca crassidens*	−	−	−	19 April, 2018	Qingdao

**TABLE 2:** Detection rate of PV-HMU-2 among belugas from Dalian Ocean Park.

Numbers	Animal species	Throat swab	Anal swabs	Sampling date	Sampling place
1	*Delphinapterus leucas*	Null	−	18 April, 2018	Dalian
2	*D. leucas*	+	Null	18 April, 2018	Dalian
3	*D. leucas*	+	−	18 April, 2018	Dalian
4	*D. leucas*	−	Null	18 April, 2018	Dalian
5	*D. leucas*	−	−	18 April, 2018	Dalian
6	*D. leucas*	+	−	18 April, 2018	Dalian
7	*D. leucas*	+	Null	18 April, 2018	Dalian
8	*D. leucas*	+	−	18 April, 2018	Dalian

### 2.2. Viral nucleic acid library construction

Samples from each species were combined by adding 1 mL from each sample into one fresh sample tube. Based on the swab sample locations, the 21 samples were divided into two pools and passed through 0.45 μm filters (Millipore Sigma, Burlington, MA, USA) to remove eukaryotic cells and large bacteria. The filtrate was then ultracentrifuged at 100,000 × *g* at 4°C for 3 h. The precipitates collected from the two pool samples were resuspended in 100 μL Hank’s balanced salt solution and digested using DNase, which consisted of 14 U of TURBO DNase (Ambion, USA), 20 U of benzonase (Novagen, Germany), and 20 U of RNase One (Promega, USA) at 37°C for 2 h in 1 × DNase buffer to decompose and remove unprotected nucleic acids. Viral DNA and RNA were extracted using QIAamp Viral RNA Mini Kit (Qiagen, Hilden, Germany), according to the manufacturer’s instructions. First-strand cDNA was generated using Superscript III Reverse Transcriptase (Invitrogen, Thermo Fisher Scientific), as previously described (Wu et al., 2018b). Double-stranded DNA was synthesized using Klenow fragment enzyme combined with K-8N (GACCATCTAGCGACCTCCACNNNNNNNN). To eliminate excess enzymes and PCR product primers, a PCR purification kit was used, followed by the use of magnetic beads to adsorb 300–2,000 bp fragments and dissolve them in nuclease free water. The nucleic acid library was then constructed using the DNA Library Prep Kit (Invitrogen Collibri, USA).

### 2.3. Next-generation sequencing

The amplified viral nucleic acid libraries were sequenced with an Illumina HiSeq 2500 sequencer using the 150 bp paired-end method (Illumina Inc., San Diego, CA, USA). To maximize the available length and total output of the raw data, each pool sample library was sequenced with separate channels and indexing. The raw sequence reads were filtered, and valid sequences were obtained using previously described criteria. The initial image analysis and base calling were performed using the GAPipeline program with default parameters. Sequences that include the primer K and adaptor are extracted using the Illumina filter (Wu et al., 2012). The sequence data were deposited into the National Center for Biotechnology Information sequence read archive under the accession number PRJNA650224.

### 2.4. Taxonomic assignment

Raw sequence reads were filtered to obtain valid sequences using previously described criteria (Yang et al., 2011). Assembly of quality-controlled reads of each sample into contigs was performed with Trinity (Version 2.5.1). Diamond (Version 0.9.14.115) software was used to translate contigs into amino acid sequences. Sequence similarity-based taxonomic assignments were conducted as described previously (Yang et al., 2011). Briefly, each sequence was aligned with the NCBI non-redundant protein databases (NR) using the basic local alignment search tool (Blast)^1^ Blast X (E-value < 10^–5^, −F: filter query sequence, default = T) to determine its viral origin. All Blast X results of contigs were annotated with taxonomy IDs MegaHit and other information obtained from the NCBI Entrez server (Tamura et al., 2013).

### 2.5. Genome sequencing of papillomavirus

Molecular clues obtained from metagenomic analyses were utilized to classify sequence reads into viral families or genera using MEGAN.^2^ Representative reads of novel papillomaviruses were selected for PCR amplification and sequencing verification, and the complete novel PVs sequences were amplified. To amplify and sequence parts of the genome, specific nested PCR primers were designed. cDNA was generated using Superscript III Reverse Transcriptase (Invitrogen/United States) and random primers. All primer sequences were based on newly obtained reads and amplified sequences, and the primer sequences used are shown in Supplementary Table 2.

### 2.6. Genome annotation

The viral nucleotide sequences of the genomes and the amino acid sequences of the ORFs were deduced by comparing them against those of other viral families. Conserved protein families and domains were predicted using Pfam,^3^ Blast p (see text footnote 1), and InterProScan 5 (see text footnote 3). The genome structure diagram was constructed using IBS 1.0.3 software, and routine sequence alignment was performed using Clustal Omega.^4^ TFBS was predicted with “BDGP”^5^ and “Softberry.”^6^

### 2.7. Calculation of viral prevalence

Specific primers targeting the non-structural gene were designed based on the partial viral genomic sequences obtained from NGS for PV screening in each individual sample (Supplementary Table 2). PCR was performed using GoTaq Colorless Master Mix (Promega, Madison, WI, USA). The first-round PCR product (2 μl) as the template for the second round of PCR was used to achieve high specificity and sensitivity. The thermal cycling conditions for PCRs were as follows: 94°C for 5 min, followed by 35 cycles of 94°C for 30 s, 57°C for 35 s, 72°C for 30 s, and a final elongation step at 72°C for 10 min. The PCR products were analyzed using 1.5% agarose gel electrophoresis and ultraviolet imaging (Bio-Rad, USA).

### 2.8. Phylogenetic analysis

An amino acid alignment was performed between the novel beluga PV and other reference sequences using the Blast tool on the NCBI to identify closely related sequences. The phylogenetic tree included both mammalian and non-mammalian papillomaviruses, and representative species from each genus were selected as reference sequences. According to a previous study, each PV reference sequence is concatenated independently (Agius et al., 2019). A total of 61 PVs reference sequences were combined with the novel PVs sequences in this study to construct a phylogenetic analysis tree. MEGAX was used to align the amino acid sequences using the MUSCLE package with default parameters. The phylogenetic tree was constructed using the maximum likelihood method. The best substitution models were the Poisson model with uniform rates among sites, and this model was replicated with 1,000 bootstrap replicates to construct the phylogenetic tree.

## 3. Results

### 3.1. Discovery of two novel PVs in belugas using NGS

**FIGURE 1:**
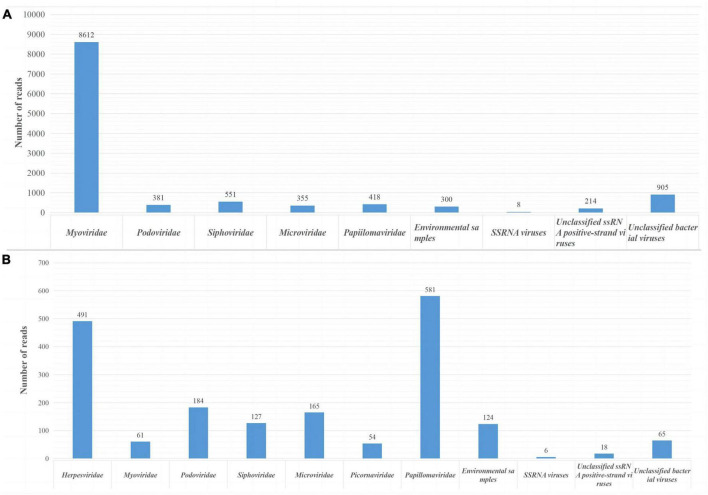
Viral reads from two sample pools using next-generation sequencing (NGS). **(A)** Identification of viral groups in swab samples from Qingdao belugas and false killer whale. **(B)** Viral content in swab samples from Dalian belugas.

In this study, we obtained 2.74 GB (214,466 valid reads, 150 bp in length) and 1.39 GB (102,834 valid reads, 150 bp in length) of nucleotide data from sample pools 41 and 49, respectively. Sequences of less than 50 bp and duplicate sequences were eliminated. The archaea, bacteria, microbial eukaryotes, and those that had no significant similarity with any amino acid sequences in the viral NR protein database were eliminated. In pool 49, 14,055 virus-associated reads, accounting for 6.55% of the total reads, were identified, while in pool sample 41, 2,404 virus-associated reads, accounting for 2.33% of the total reads were identified. The viromes of both pools consisted of double-stranded (ds) DNA, single-stranded (ss) DNA, ssRNA, and retro-transcribing viruses. The virus-associated reads of two pool samples were assigned to families such as *Picornaviridae*, *Herpesviridae*, *Myoviridae*, *Podoviridae*, *Siphoviridae*, *Microviridae*, *Retroviridae*, and unclassified viruses (Figure 1). Moreover, swab sample pools from Qingdao and Dalian contained 418 and 54 papillomavirus reads, respectively, with PVs accounting for 2.97 and 2.24% of the total viruses in pools 49 and 41, respectively. After PCR verification, two novel PV sequences were obtained and compared with the original sequencing PV reads, which showed that the PV reads in the metagenomic sequencing results belonged to the two novel PVs. In addition, molecular clues related to mammalian *Picornaviridae* and *Herpesviridae* were found in the whale swab samples from Dalian, with 491 reads related to herpesviruses showing less than 50.68% aa identity with other known herpesviruses and being related to Beluga alpha herpes virus 1. Furthermore, the sequencing results showed 56 reads related to picornavirus, with less than 40.24% aa identity with other known picornaviruses, and verification of this virus was completed by our laboratory (Wang et al., 2021).

### 3.2. Genomic characterization of novel PVs

**FIGURE 2:**
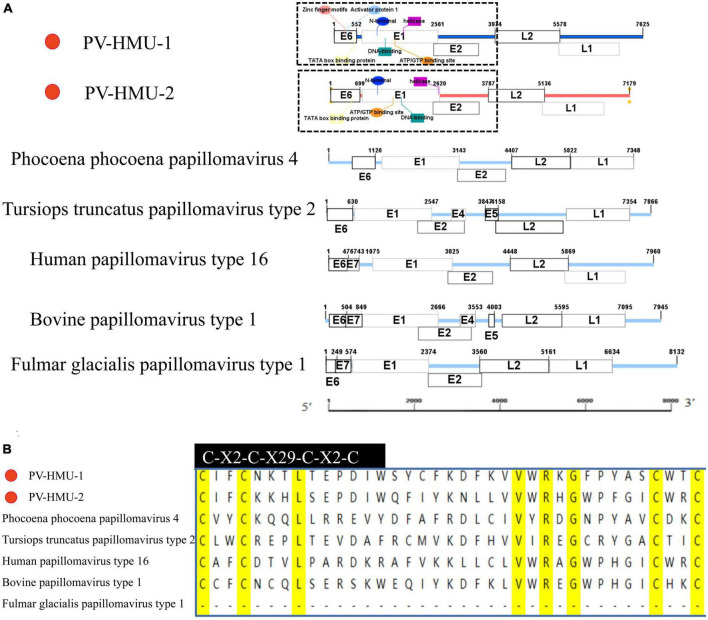
Sequence and motif annotation of PV-HMU-1 and PV-HMU-2. **(A)** Putative and confirmed early and late ORFs identified in PV-HMU-1 and PV-HMU-2 genomes using ORF predictor. We used IBS 1.0.3 software to construct the genomic structure diagram of PV-HMU-1, PV-HMU-2, *Phocoena phocoena* papillomavirus 4 (PphPV4) and *Lagenorhynchus acutus* papillomavirus (GU117624). **(B)** The E6 conserved protein domains of PV-HMU-1 and PV-HMU2 (light blue shade) was aligned with other papillomaviruses. The C-terminal zinc-binding domains for PV-HMU-1, PV-HMU-2, HPV16, BPV1, TtPV2, and PphPV4 are indicated.

Specific primers were designed based on the NGS-generated fragment sequences to obtain the complete genome sequences of two novel PVs from samples obtained from belugas in Qingdao and Dalian Ocean Parks. The novel PVs were named PV-HMU-1 (GenBank accession: OP748391) and PV-HMU-2 (GenBank accession: OP748392), respectively. The complete genome length of PV-HMU-1 and PV-HMU-2 were 7,624 and 7,179 bp, respectively, with C + G contents of 58.77 and 54.37% (Figure 2 and Supplementary Table 1). The Pfam software predicted that the observed mammalian PV genomic structures included a core set of early (E6, E1, and E2) and late (L2 and L1) open-reading frames (ORFs) (Figure 2). ORF prediction software revealed that PV-HMU-1 E6 (184 aa) was 552 nt (1–552), E1 (625 aa) was 1,875 nt (687–2,561), early E2 (393 aa) was 1,179 nt (2,506–3,684), late L2 (535 aa) was 1,605 nt (3,974–5,578), and late L1 (582 aa) was 1,746 nt (5,337–7,082) in length. For PV-HMU-2, E6 (233 aa) was 699 nt (1–699), E1 (622 aa) was 1,866 nt (755–2,620), early E2 (372 aa) was 1,116 nt (2,556–3,671), late L2 (450 aa) was 1,350 nt (3,787–5,136), and late L1 (496 aa) was 1,488 nt (5,120–6,607) in length.

A study by Schulz et al. (2012) used TFBS prediction to identify potential *cis-*regulatory elements in the upstream regulatory region (URR) of rodent PVs. The URR sequences of PV-HMU-1 contained important TFBS, such as activator protein 1 (AP1), which were located directly upstream of the TFIID BS (TATA box binding protein). However, the TFBS of PV-HMU-2 was not clearly predicted. Both PV-HMU-1 and PV-HMU-2 included a zinc-finger motif (C-X2-C-X29-C-X2-C) in E6, while E7 was absent (Figure 2). An adenosine triphosphate/guanosine triphosphate (ATP/GTP) binding site (G-X4-GKS) was present within the helicase domain of E1 in both sequences. The N-terminal, DNA-binding domain, and helicase were located at positions 689–1,058 bp| 755–1,124 bp, 1,235–1,652 bp| 1,273–1,700 bp, and 1,707–2,504 bp| 1,712–2,589 bp of the E1 region for PV-HMU-1 and PV-HMU-2, respectively. Comparing the L2 and L1 amino acid sequences of PV-HMU-1 and PV-HMU-2 with those of other papillomaviruses, PV-HMU-1 showed 41.97 and 64.99% identity to *Phocoena* papillomavirus 4 (NC 018076), respectively, while PV-HMU-2 showed 49.19 and 68.12% identity to Sus scrofa papillomavirus 1 (MT078972), respectively.

### 3.3. Phylogenetic analyses

**FIGURE 3:**
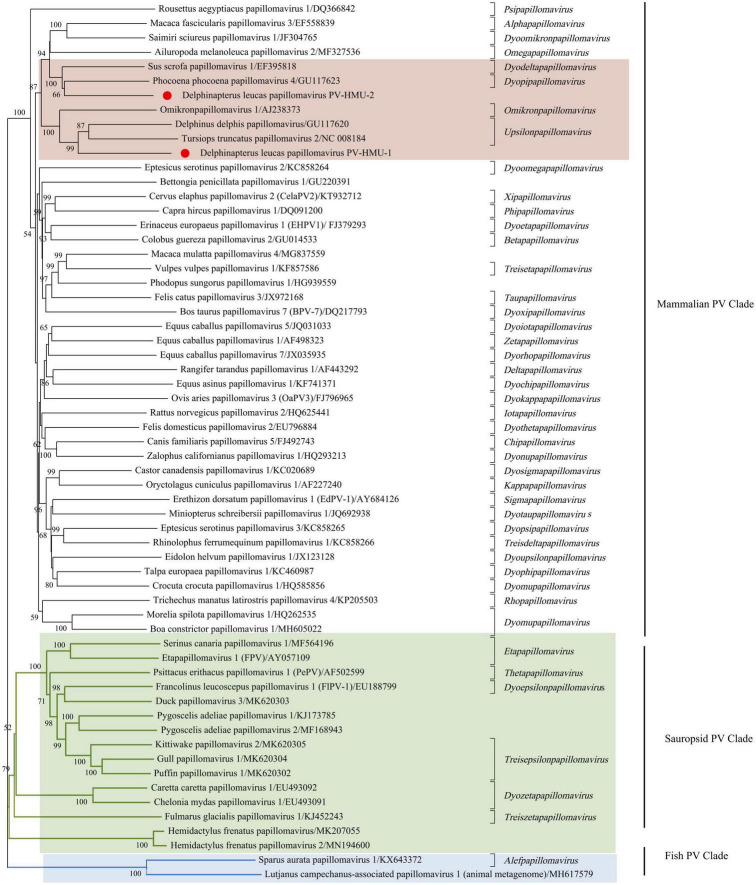
Phylogenetic tree of concatenated early (E1, E2) and late (L1) papillomavirus proteins. The evolutionary history was inferred from 61 amino acid sequences, including the novel PV-HMU-1 and PV-HMU-2, using a maximum likelihood approach. A 1,000 bootstrap replication were used to construct the tree. PV-HMU-1 and PV-HMU-2 are marked with red circles under mammalian-related PVs. The virus strains close to the novel identified PVs are marked with pink background, Sauropsid PV, and Fish PV clades are marked with green and blue backgrounds, respectively.

Phylogenetic analyses were conducted using the complete E1-E2-L2-L1 amino acid sequences of PV-HMU-1 and PV-HMU-2, as well as 61 representative references related to *Papillomaviridae* in GenBank (Figure 3). Based on the results of Blast and preliminary evolutionary analysis, the novel PVs were attributed to the mammalian PV clade, so we mainly selected mammalian PVs as references, with a few Sauropsid and Fish PV clades selected as out-groups. More than 50 genera of PVs existed, and we selected the representative reference of each genus and the virus strains with the highest identities relative to the novel PVs to construct the evolutionary tree. The phylogenetic analysis showed that the amino acid combination of PV-HMU-1 clustered with *Tursiops truncatus* papillomavirus 2 (TtPV2) (NC 008184) and formed a relatively independent clade within the genus *Upsilonpapillomavirus*. PV-HMU-2 clustered with *Phocoena phocoena* papillomavirus 4 (PphPV4) (GU117623), the only species of *Dyopipapillomavirus*. Sus scrofa papillomavirus 1 is a member of the genus *Dyoomikronpapillomavirus* and was clustered with PV-HMU-2. PV-HMU-1 and PV-HMU-2 did not cluster on the same branch on the evolutionary tree and were far from human-associated papillomaviruses. The genome sequences of PV-HMU-1 and PV-HMU-2 carried by belugas were difficult to compare with those of other known papillomaviruses at the nucleic acid level in the NCBI nucleotide database. Genome identity and phylogenetic analyses suggested that these beluga PVs represented novel species.

### 3.4. Prevalence of PV infection in belugas

Gene-specific nested primers targeting the L1 gene region for PCR were designed based on the genomic sequences of novel PVs to screen for their presence in the throat, nose, and anal swabs. The throat and nose swabs of belugas in both locations showed a high infection rate of PVs, while the infection rate in anal swabs was low. Thirty-three percent (1/3) of throat swabs and nose swabs, but no anal swabs were positive for PV-HMU-1 (Table 1). Seventy-one percent (5/7) of throat swabs but no anal swabs were positive for PV-HMU-2 (Table 2). All samples collected in Qingdao and Dalian were collected three times in April, September, and December 2018 to observe the virus-carrying rate in different quarters. The throat swabs of three animals in Dalian consistently tested positive for PV-HMU-2 at all three sampling times, indicating persistent viral presence. To confirm the presence of the same virus infection in different months, a set of L primers was used to screen the samples. After amplification, fragments of appropriate size were obtained and sent for sequencing. The sequencing results of two positive samples collected at different times showed an aa identity of > 99%. The positive samples were identified by amplification products of 540 bp for PV-HMU-1 and 522 bp for PV-HMU-2.

## 4. Discussion

The increasing contact between humans and marine mammals due to the exploration of the oceans and interest in these animals has led to a higher risk of zoonotic diseases that can endanger human health. While efforts have been made to characterize novel PV types in different animal hosts, knowledge about PV diversity in marine mammals remains limited. In recent years, many viruses, including picornaviruses, coronaviruses, morbilliviruses, and poxviruses, have been detected in dolphins (Robles-Sikisaka et al., 2012), seals (Fouchier et al., 2004), and sea lions (Waltzek et al., 2012). Due to the continuous expansion of the host range of viruses in marine mammals, it is suggested that they may serve as important hosts for many viruses, and there may still be many unknown viruses in these hosts. Reports of marine mammals acting as reservoirs for viruses are increasing. In Groth et al. (2014) obtained a complete sequence of influenza A virus (H13N2) from a sick pilot whale, while Sacristán et al. (2019) and Vargas-Castro et al. (2021) found that dolphins in the South Atlantic and whales in Spain were highly infected with herpesvirus (Sacristán et al., 2019; Vargas-Castro et al., 2021). Beluga coronavirus was detected in a study at the Washington University School of Medicine (Mihindukulasuriya et al., 2008), and a novel bottlenose dolphin coronavirus was detected at the University of Hong Kong (Woo et al., 2014). Changes in living habits, social behaviors, and migration patterns are significant factors in the transmission and spread of viruses among cetaceans. Monitoring the viruses carried by marine mammals is crucial for preventing potential infectious diseases of cetacean origin. In this study, we detected PV-HMU-1 and PV-HMU-2 in belugas from Qingdao and the Dalian Polar Ocean Parks and obtained their complete genomes. The genomes of PV-HMU-1 and PV-HMU-2 contain five ORFs (E6, E1, E2, L2, and L1) of mammal PVs but do not have the E7 ORF typical of *Alphapapillomavirus* (Figure 2). The E6 protein of the novel virus contains a zinc-finger motif that is crucial for the virus to invade the cell environment. AP1 is present at the PV-HMU-1 TFBS site and is used to regulate α-HPV expression. The two novel PVs contain an ATP/GTP binding site, an N-terminal, DNA binding region, and a helix in the E1 region. Papillomaviruses are generally transmitted through sex, close contact, and mother-to-child transmission. The 50% infection rate of PV-HMU-2 infection in individual samples reflected the possibility of a high incidence of PV infection in belugas. Belugas that tested positive for PV-HMU-1 and PV-HMU-2 were positive three times within a year, indicating that the infection was persistent. It is important to note that some of the negative results may be due to inadequate sampling.

In 2019, the ICTV report on the classification of genera highlighted that, based on multiple sequences or comparative pairs of L1 genes, most PV genus types have less than 60% sequence identity with other types, but this is not the only criterion (de Villiers et al., 2004). The L gene aa identity between PV-HMU-1 and PV-HMU-2 was 65.21%, although they were found in the same beluga host. The two novel PVs showed less than 68.12% aa identity with the L gene region of known papillomaviruses. The new viruses mainly cluster with viruses whose hosts are small toothed whales in the phylogenetic tree, which are closely related to *Upsilonpapillomavirus* and *Dyopipapillomavirus*. The clustering of PV-HMU-2 and pig PV suggests that the evolution of PV in belugas and terrestrial animals is related.

Several phylogenetic studies have shown that a complex evolutionary scenario drives PV diversity, such as recombination, co-divergence with the host, interspecies transmission, and adaptation to new environments (Geoghegan et al., 2017). Although the belugas in this study did not exhibit obvious tumors or other clinical manifestations, the high infection rate suggests a possible association between papillomavirus infection and belugas. While this study amplified the complete genome sequences of two PVs and investigated their classification, it is merely a small step toward understanding the complexities of papillomaviruses in marine mammal populations. More work is necessary to examine the evolution, transmission, and biological role of papillomaviruses in marine mammal populations, isolate and record them, and provide fundamental data for future research on the evolution, classification, and traceability of mammalian viruses.

In this study, we reported the discovery of two novel papillomaviruses in the throat and nasal swabs collected from belugas in Qingdao and Dalian. To our knowledge, belugas have never been reported as hosts for papillomaviruses before. These findings increase our understanding of the mammal-borne virus community in Qingdao and Dalian and provide preliminary data for future research on the evolution of PVs. However, further investigation is necessary to determine the pathogenicity of these two novel viruses and their potential impact on both humans and animals.

## Data availability statement

The datasets presented in this study can be found in online repositories. The names of the repository/repositories and accession number(s) can be found in this article/Supplementary material.

## Author contributions

FY, JD, and PZ designed the study. PZ, SB, XS, and XL collected the specimens. YL, ZL, MX, YZ, GW, RP, HS, and XH performed the experiments. CT and GL analyzed the data. YL wrote the draft manuscript. JD and PZ edited the manuscript. All authors read and approved the final manuscript.

## Footnotes

1
https://blast.ncbi.nlm.nih.gov

2
https://megasoftware.net/show_eua

3
http://www.ebi.ac.uk/services/proteins

4
http://www.ebi.ac.uk/tools/

5
http://www.fruitfly.org/seq_tools/promoter.html

6
http://www.softberry.com/
